# The Cost of Drugs to Hospitals

**Published:** 1905-10-07

**Authors:** 


Vol. XXXIX.—No. 993. SATURDAY, OCTOBER 7, 1905.

### THE COST OF DRUGS TO HOSPITALS.

One of the numerous questions which arise in connection with hospital management is the method to be adopted for securing a proper supply of drugs and medicines. At first sight the matter might appear to be a purely economic one and capable of settlement by a commercial bargain arranged by free competition in the open market. Food and other necessaries, it may be urged, are purchased in this way, why not also drugs? Such a statement has a highly plausible sound and is apt to be regarded as conclusive by those who are concerned chiefly with the commercial side of hospital management. The difficulty in accepting such a position is provided by the necessity of obtaining adequate security that the medicines supplied are of such a quality that their efficiency may be taken for granted. It is manifest that whilst the quality of meat, bread, sugar, and other food stuffs is readily determined by anyone possessed of common sense and a moderate experience, medicinal preparations require the verdict of an expert. Further, it is generally allowed that whilst such a verdict is easily obtained in regard to chemical substances capable of submission to exact analytical tests, a very different position exists in the case of the large and important group of preparations technically known as galenicals. The exact medicinal value of many individual tinctures, infusions, extracts, etc., does not depend on one or more chemical principles the percentage of which may be definitely estimated. Hence no laboratory standard for such preparations exists, and as a matter of fact the pharmacological values of different specimens of one and the same preparation vary within wide limits, nor, until they are subjected to the final medicinal test, can such specimens be distinguished one from the other by any known laboratory method. In short, the preparations now under consideration have in ordinary commercial transactions to be accepted as genuine mainly on the guarantee of the name and reputation of the firm by which they are manufactured. When this position is fully appreciated, and when, further, it is recalled how vitally important in the interests of many individual patients is a supply of pure and active medicines, it becomes obvious that the question of drug supply does not accurately adapt itself to the economic rule which governs most of the transactions in which hospital managers find themselves engaged.

From all this there arises the obvious suggestion that a hospital should manufacture pharmaceutical preparations for itself. The specimens of crude drugs, leaves, barks, roots, etc., as met with in the open market vary doubtless in quality, but their value is capable of fairly accurate appreciation by expert judgment. If this is granted it follows that from properly selected specimens medicinal preparations of reliable strength and value could be manufactured in the hospital pharmaceutical laboratory. This would afford a direct and personal guarantee that such preparations were actually what they profess to be, and this position does not seem to be capable of certain attainment by any other method. But any such proposal cannot possibly escape examination on its economic side. Desirable as absolute certainty of the strength of medicinal preparations doubtless is, hospital finances are rarely in such a position as to permit either here or elsewhere unqualified pursuit of the ideal. Pounds, shillings, and pence must receive due recognition. This we fully recognise, and we also allow that at present an exact basis of figures does not exist on which may be framed a close comparison between the expense involved in the purchase of medicinal preparations on the one hand, and their manufacture on the other. But whilst admitting all this we desire to urge that, as articles published in this and in a previous issue show, there is no justification for concluding *a priori* that for a hospital to produce its own medicinal preparations is an economic mistake. The question requires further exact estimates and calculations, but as our contributor shows, the manufacturing claim is not without force. In addition to mere ledger calculations it may be suggested that whatever makes hospital pharmacy expert and scientific has prospects of economy in many directions, and a highly capable

192722

pharmacist may save his hospital much wasteful expenditure. Further, the argument is not concluded even if the economic position should oppose a pharmaceutical laboratory in the individual hospital. Is not combination possible? and may not hospitals unite both to secure food and other supplies, and also to manufacture medicinal preparations for their own needs? This may conceivably be the solution of many problems which agitate the hospital world at the present moment.

